# Efficacy of adjunctive inhaled colistin and tobramycin for ventilator-associated pneumonia: systematic review and meta-analysis

**DOI:** 10.1186/s12890-024-03032-7

**Published:** 2024-05-02

**Authors:** Jefferson Antonio Buendía, Diana Guerrero Patiño, Andrés Felipe Zuluaga Salazar

**Affiliations:** 1 Research Group in Pharmacology and Toxicology, Department of Pharmacology and Toxicology, University of Antioquia, Medellín, Colombia; 2 Division of Health Sciences, Warwick Medical School, University of Warwick, Coventry, UK; 3 Laboratorio Integrado de Medicina Especializada (LIME), Facultad de Medicina, IPS Universitaria, Universidad de Antioquia, Antioquia, Colombia; 4 Facultad de Medicina, Universidad de Antioquia, Carrera 51D #62-29, Medellín, Colombia

**Keywords:** Ventilator-associated pneumonia, Efficacy, Safety

## Abstract

**Introduction:**

Ventilator-associated pneumonia (VAP) presents a significant challenge in intensive care units (ICUs). Nebulized antibiotics, particularly colistin and tobramycin, are commonly prescribed for VAP patients. However, the appropriateness of using inhaled antibiotics for VAP remains a subject of debate among experts. This study aims to provide updated insights on the efficacy of adjunctive inhaled colistin and tobramycin through a comprehensive systematic review and meta-analysis.

**Methods:**

A thorough search was conducted in MEDLINE, EMBASE, LILACS, COCHRANE Central, and clinical trials databases (www.clinicaltrials.gov) from inception to June 2023. Randomized controlled trials (RCTs) meeting specific inclusion criteria were selected for analysis. These criteria included mechanically ventilated patients diagnosed with VAP, intervention with inhaled Colistin and Tobramycin compared to intravenous antibiotics, and reported outcomes such as clinical cure, microbiological eradication, mortality, or adverse events.

**Results:**

The initial search yielded 106 records, from which only seven RCTs fulfilled the predefined inclusion criteria. The meta-analysis revealed a higher likelihood of achieving both clinical and microbiological cure in the groups receiving tobramycin or colistin compared to the control group. The relative risk (RR) for clinical cure was 1.23 (95% CI: 1.04, 1.45), and for microbiological cure, it was 1.64 (95% CI: 1.31, 2.06). However, there were no significant differences in mortality or the probability of adverse events between the groups.

**Conclusion:**

Adjunctive inhaled tobramycin or colistin may have a positive impact on the clinical and microbiological cure rates of VAP. However, the overall quality of evidence is low, indicating a high level of uncertainty. These findings underscore the need for further rigorous and well-designed studies to enhance the quality of evidence and provide more robust guidance for clinical decision-making in the management of VAP.

**Supplementary Information:**

The online version contains supplementary material available at 10.1186/s12890-024-03032-7.

## Background

Ventilator-associated pneumonia (VAP) poses a significant challenge in intensive care units (ICUs) [1]. The rise in drug-resistant gram-negative pathogens, such as extended-spectrum beta-lactamase (ESBL)-producing and carbapenem-resistant Enterobacteriaceae, has been associated with treatment failures and increased mortality in ICU patients [2]. To address this issue, the use of adjunctive inhaled antibiotics has emerged as a viable strategy [3]. Inhaled antibiotics offer the advantage of achieving higher drug concentrations in the pulmonary epithelial cells, which is particularly beneficial for treating gram-negative infections with aminoglycosides [4]. Current clinical guidelines recomended to use inhaled antibiotics in addition to intravenous therapy, especially for patients infected with colistin/aminoglycoside-sensitive pathogens or those who have shown a poor response to intravenous antibiotics [5]. This approach improves treatment success rates while minimizing systemic antibiotic doses and associated toxicities.

Colistin and tobramycin are the most commonly prescribed nebulized antibiotics [6]. Previous systematic reviews have examined the efficacy of adjunctive inhaled antibiotic therapy for VAP treatment [7–10]. However, contrary to more recent studies, these systematic reviews did not find any advantages in utilizing adjunctive inhaled antibiotics, including in terms of clinical cure rates. Furthermore, differing opinions among experts have raised concerns regarding the appropriateness of prescribing inhaled antibiotics for patients with VAP [11]. This study presents a comprehensive systematic review and meta-analysis, offering updated insights on the effectiveness of adjunctive inhaled colistin and tobramycin.

## Methods

### Search strategy

We conducted a comprehensive search across multiple databases, including MEDLINE, EMBASE, LILACS, and COCHRANE Central, to identify relevant studies. Additionally, we explored clinical trial databases such as www.clinicaltrials.gov, www.base-search.net/, www.tripdatabase.com/, preprinted servers (MedRxiv, JMIR Preprints) and thesis and dissertations (Dart-Europe, EThOs, https://oatd.org/). The search spanned from the inception of the databases to June 2023. We also manually searched the reference lists of eligible studies for additional relevant articles. No language restrictions were applied. The detailed search strategy can be found in the Supplemental material.

### Outcomes

This study aimed to evaluate the effectiveness of inhaled Colistin and Tobramycin on clinical cure (primary outcome) and in-hospital mortality, microbiological Cure, and incidence of adverse events (secondary outcomes). For this review, we adopted the definition of clinical and microbiological cure as provided by each individual study. Mortality was defined as death from any cause within 30 days of initiating the intervention.

### Inclusion criteria

Randomized controlled trials meeting specific criteria were included in our analysis. The criteria encompassed the following: 1) the study population consisted of mechanically ventilated patients diagnosed with ventilator-associated pneumonia (VAP); 2) the intervention involved the use of inhaled Colistin and Tobramycin for the treatment of VAP, compared to intravenous antibiotics; and 3) the study reported at least one of the following outcomes: clinical cure, microbiological eradication, mortality, or adverse events. Articles that did not fulfill all of these criteria relating to the population, intervention, comparison, and outcome of interest were excluded. Furthermore, review conferences, letters, commentaries, non-randomized controlled trials, and animal experimental studies were also excluded.

### Study selection and data extraction

Two independent reviewers performed the selection of studies and extraction of data. Screening of titles and abstracts was conducted based on the predefined inclusion criteria. Full-text articles were obtained for studies that met the inclusion criteria, while articles that did not meet these criteria were excluded. Disagreements between reviewers were resolved through consensus.

### Risk of bias assessment

Two reviewers assessed the risk of bias (RoB) of the included studies with the Cochrane RoB tool [12]. Disagreements were resolved by consensus. The risk of publication bias among the studies was planned to be assessed by visual inspection of the funnel plot figure. To evaluate the quality of the included literature, and the GRADE tool (GDT) was used to evaluate the quality of the included outcomes [13].

### Data synthesis and statistical methods

For dichotomous outcomes, we calculated relative risk (RR) with their 95% confidence interval (95%CI). Heterogeneity was assessed using the I^2^ statistics calculated from Cochran’s Q test. Since we recognise that the studies are based on multiple populations, we chose to use the random-effects model for the analysis, regardless of the I^2^ results. All statistical analysis was performed using Review Manager (RevMan 5.4).

## Results

**Fig. 1:**
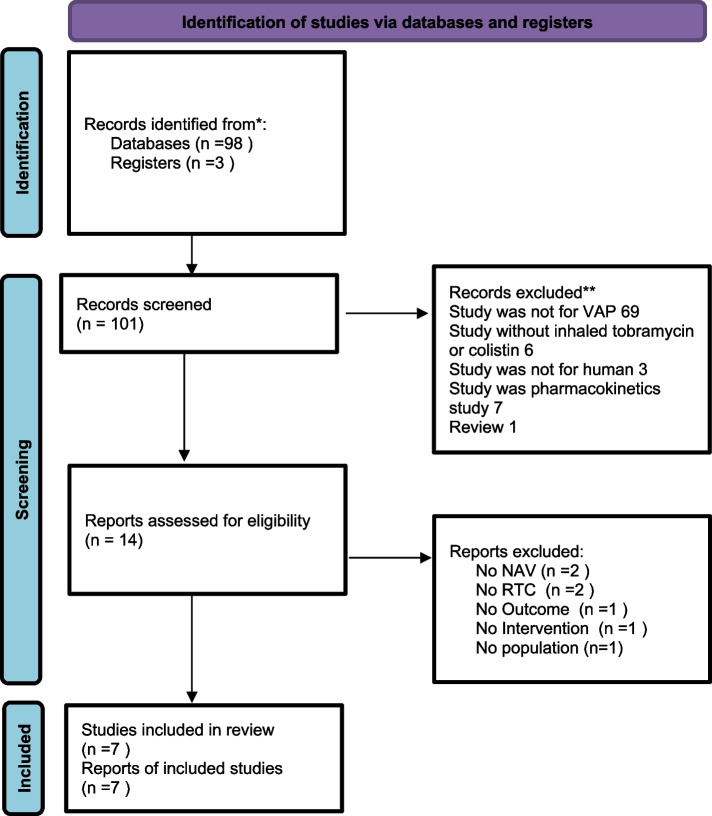
PRISMA 2020 flow diagram of identificacion of studies in the systematic review

The initial search yielded 106 records, which were subsequently reduced to 31 after removing duplicates. Following the screening of titles and abstracts, 59 records were excluded, as shown in Fig. 1. Consequently, only seven randomized controlled trials (RCTs) that met the predefined inclusion criteria were included in the meta-analysis [14–20].

Comprehensive information regarding the included studies is provided in the Supplementary material. The studies range in design, with sample sizes varying from smaller studies like Hallal with 10 participants (5 treatment, 5 control) to larger studies such as Rattanaumpawan, which included 100 participants (51 treatment, 49 control) [14–18, 20]. In these studies, a broad range of patient ages were included. Each study employed specific diagnostic criteria for VAP and reported a diverse distribution of bacterial species responsible for infections. For example, Rattanaumpawan focused on patients with Gram-negative bacteria isolated from an endotracheal tube aspirate, including Acinetobacter (50%) and Pseudomonas (26%), among others [19]. The distribution of various bacterial species causing VAP was also diverse across the studies, with Pseudomonas aeruginosa, Acinetobacter spp., and Klebsiella spp. being some of the most reported pathogens. Specific interventions included the use of tobramycin at dosages like 40 mg every 8 h in the case of Brown, and colistin nebulized at 75 mg every 12 h for a duration of 9 to 12 days in Rattanaumpawan. These interventions were compared against control groups that received placebo treatments or systemic antibiotics without the aerosolized form. The duration of the interventions varied, with some studies specifying the exact number of days the aerosolized antibiotics were administered and others not. For instance, Le Conte employed tobramycin for 5 days, whereas Nassar did not provide specific duration details for the use of colistin [17, 18].

Table 1 presents the biases observed in these studies, indicating that three studies exhibited attrition bias, one had reporting bias, two had detection bias, and one had performance bias. The GRADE results, as depicted in Table 2, indicated a “very low quality” of evidence for both clinical cure and adverse events. Due to the limited number of studies, the interpretation of the results is constrained, and it is difficult to ascertain the risk of publication bias with sufficient confidence.

**Table 1:**
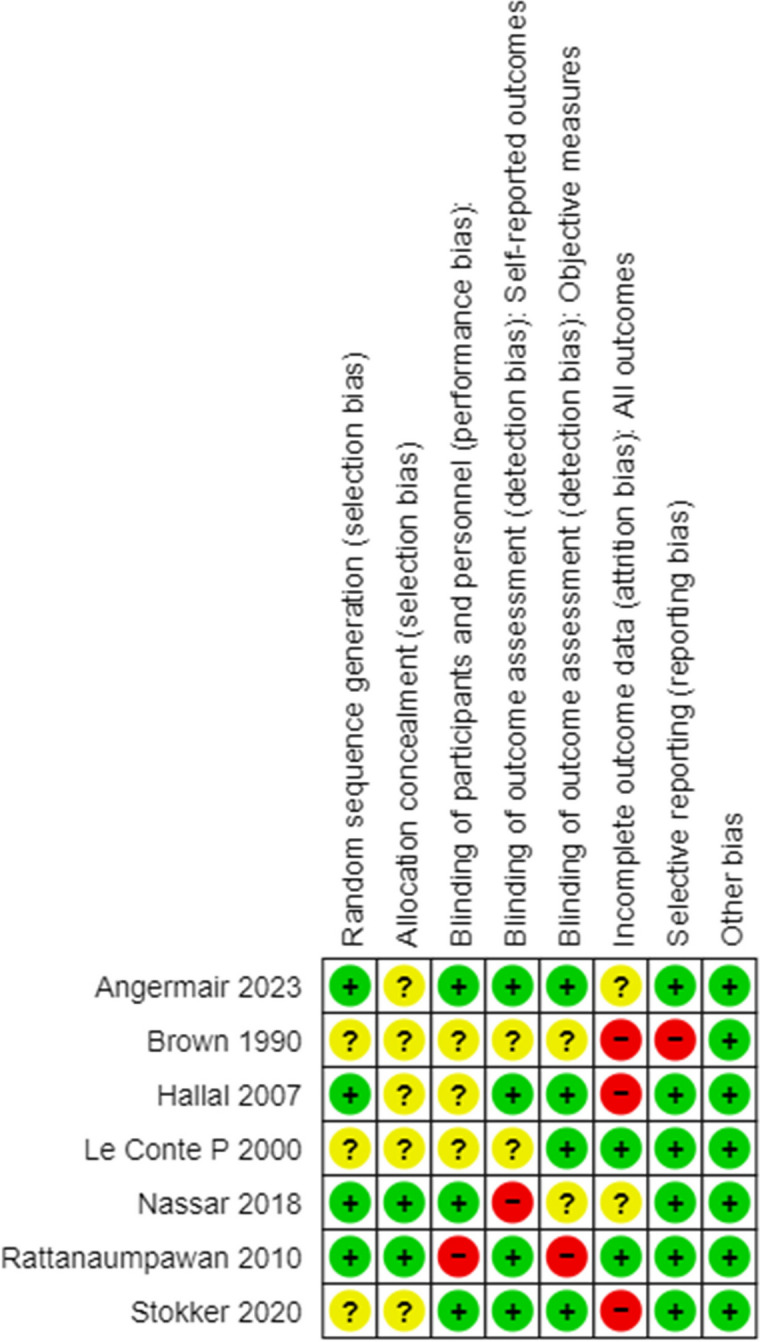
Risk of bias

**Table 2:** Results of quality of the included outcomes by GRADE tool

Colistin and tobramycin compared to control for ventilator-associated pneumonia					
**Outcomes**	**№ of participants (studies) Follow-up**	**Certainty of the evidence (GRADE)**	Relative effect (95% CI)*	**Anticipated absolute effects**	
				**Risk with control**	**Risk difference with Colistin and Tobramycin**
Clinical cure	242 (7 RCTs)	⨁◯◯◯ Very low^a,b,c^	**RR 1.23** (1.04 to 1.45)	512 per 1,000	**118 more per 1,000** (20 more to 230 more)
Microbiological cure	279 (5 RCTs)	⨁⨁⨁◯ Moderate^a^	**RR 1.64** (1.31 to 2.06)	432 per 1,000	**276 more per 1,000** (134 more to 458 more)
All cause mortality	333 (6 RCTs)	⨁⨁◯◯ Low^a,c^	**RR 0.82** (0.59 to 1.14)	452 per 1,000	**81 fewer per 1,000** (185 fewer to 63 more)
Adverse events	152 (3 RCTs)	⨁◯◯◯ Very low^a,c,d^	**RR 1.31** (0.75 to 2.28)	216 per 1,000	**67 more per 1,000** (54 fewer to 277 more)

GRADE Working Group grades of evidence

High certainty: we are very confident that the true effect lies close to that of the estimate of the effect

Moderate certainty: we are moderately confident in the effect estimate: the true effect is likely to be close to the estimate of the effect, but there is a possibility that it is substantially different

Low certainty: our confidence in the effect estimate is limited: the true effect may be substantially different from the estimate of the effect

Very low certainty: we have very little confidence in the effect estimate: the true effect is likely to be substantially different from the estimate of effect

*CI* Confidence interval, *RR* risk ratio

^*^The risk in the intervention group (and its 95% confidence interval) is based on the assumed risk in the comparison group and the relative effect of the intervention (and its 95% CI)

Explanations

^a^The level of heterogeneity was more than 25% and was not explained completely, therefore, there was serious inconsistency

^b^Although double blinding was not conducted in two studies (Rattanaumoawan P and Nassar YS) and was unclear in one study (LeConte P), no risk of bias was detected for mortality

^c^Funnel plot asymmetry

^d^Small sample size and few studies

### Meta-analysis of outcomes

#### Clinical cure

**Fig. 2:**
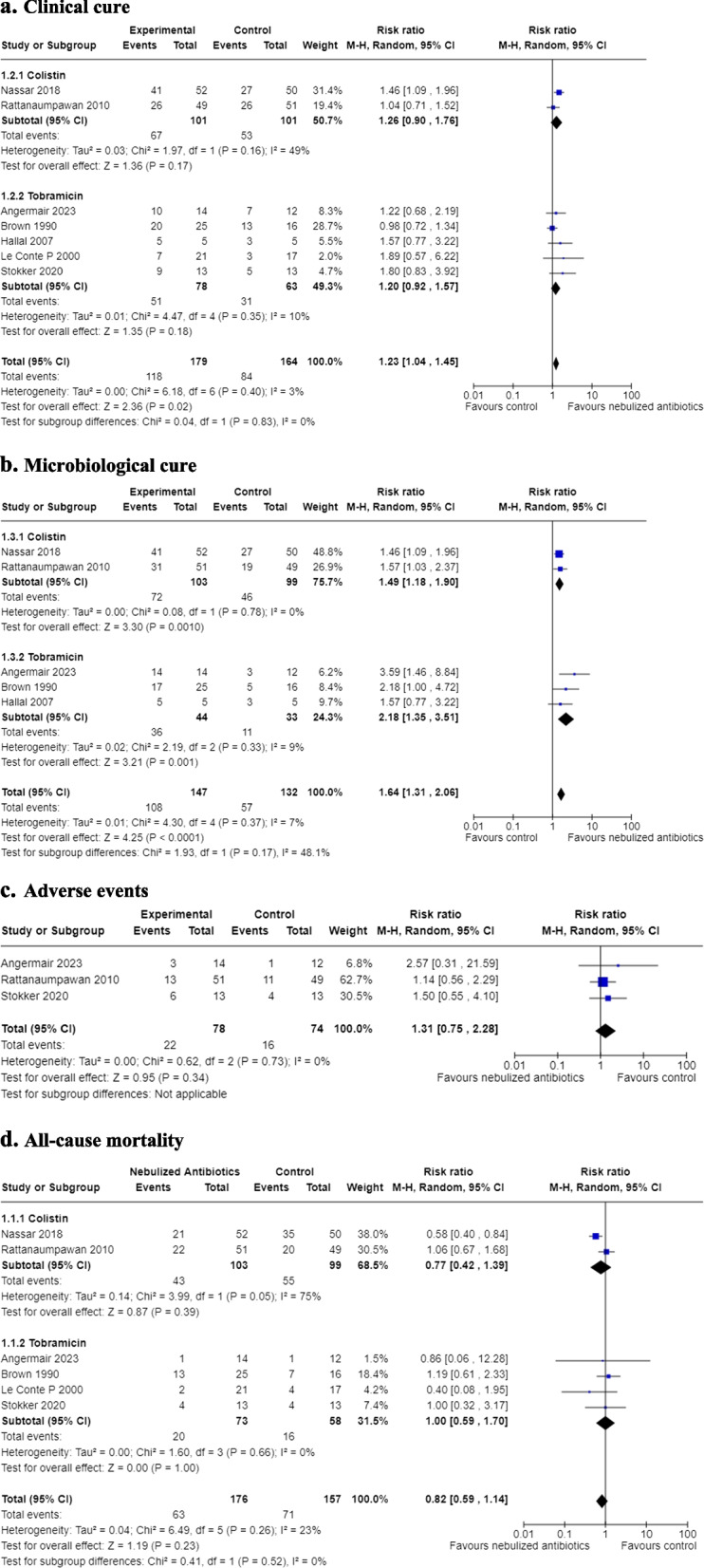
Forest plot of principal outcomes. **a** Clinical cure. **b** Microbiological cure. **c** Adverse events. **d** All-cause mortality

The meta-analysis included a total of seven RCTs, comprising 242 patients. The analysis revealed a higher probability of achieving clinical cure in the groups receiving tobramycin or colistin compared to the control group, with a relative risk (RR) of 1.23 and a 95% confidence interval (CI) of (1.04, 1.45). However, this evidence is rated as “very low quality,” as indicated in Fig. 2a.

#### Microbiological cure

Five RCTs, involving 279 patients [2–6], were included in the meta-analysis for the outcome of microbiological cure. The results demonstrated an effect of achieving microbiological cure in the groups receiving tobramycin or colistin compared to the control group, with an RR of 1.64 and a 95% CI of (1.31, 2.06). The quality of evidence supporting this finding is rated as “moderate,” as illustrated in Fig. 2b.

#### Adverse events

Three RCTs, including 152 patients [5, 6], were included in the analysis of adverse events. The results did not show any significant differences in the risk of adverse events between the groups, with an relative risk (RR) of 0.75 and a 95% CI of (0.75, 2.86). The evidence supporting this outcome is rated as “very low quality,” as shown in Fig. 2c.

#### Mortality

The meta-analysis comprised six RCTs, encompassing 333 patients [5]. The analysis did not reveal any significant differences in mortality between the groups receiving tobramycin or colistin compared to the control group, with a relative risk (RR) of 0.82 and a 95% CI of (0.59, 1.14). The quality of evidence supporting this finding is rated as “low,” as presented in Fig. 2d.

## Discussion

The present systematic review and meta-analysis provide valuable insights into the efficacy and safety of adjunctive inhaled antibiotics for the treatment of ventilator-associated pneumonia (VAP). The findings suggest that the use of tobramycin or colistin as adjunctive inhaled antibiotics may have a positive impact on both the clinical and microbiological cure rates of VAP. The meta-analysis demonstrated a higher probability of clinical cure and a greater likelihood of achieving microbiological eradication in the groups receiving adjunctive inhaled antibiotics compared to the control group. However, it is important to note that the quality of evidence supporting these findings is rated as “very low” and “moderate,” indicating a significant degree of uncertainty. Therefore, further research is required to strengthen the evidence base and establish more definitive conclusions.

The results of our study align with previous evidence in the field [7, 21]. However, a notable difference between our study and previous systematic reviews is the inclusion of the studies conducted by Angermain and Hallal [14, 16]. These studies evaluated the effectiveness of tobramycin and reported positive results in terms of microbiological eradication and the probability of clinical cure, without observing a reduction in mortality. It is worth mentioning that due to their small sample sizes, the weight of these studies in the meta-analysis is relatively low compared to other studies, and thus our conclusions align with previous systematic reviews.

Our work has limitations. First, the small sample size (seven RCTs) restricts generalizability and raises concerns about chance findings. Additionally, potential biases identified in the included studies (attrition, reporting, detection, performance) could generate doubt on the reliability of the results. Furthermore, the low quality of evidence (“very low” for clinical cure and adverse events) highlights the need for further research before definitive conclusions can be drawn. The limited exploration of adverse events (only three studies reporting) restricts understanding of potential risks. The possibility of publication bias, given the restricted sample size, warrants caution in interpreting findings. Additionally, further investigation is needed to determine optimal dosing regimens and treatment durations for inhaled antibiotics. Furthermore, the impact of adjunctive inhaled antibiotics on specific patient subgroups and the potential development of antibiotic resistance requires further exploration. Our study has several strengths. Our search was exhaustive including gray literature and clinical trial registries. We follow the recommendations of the Cochrane collaboration and use GRADE to assess the quality of the evidence.

While this study sheds light on the potential benefits of adjunctive inhaled colistin and tobramycin for VAP, further research is crucial to optimize their use and refine clinical practice. Several key areas demand exploration: 1. Dosing and Treatment Duration: The optimal dosing regimen and treatment duration for inhaled antibiotics remain unclear. Future studies should explore a wider range of doses and durations to identify the most effective and safe approach, considering factors like patient characteristics and pathogen susceptibility. 2. Subpopulation Analysis: The present analysis did not delve into potential differences in treatment response across diverse patient subgroups (e.g., specific pathogens, underlying comorbidities). Future research should stratify analyses by relevant subgroups to provide more nuanced insights on which patients benefit most from this intervention. 3. Delivery System Optimization: The impact of delivery systems on drug bioavailability in the lungs was not addressed. Investigating and optimizing delivery systems could significantly improve treatment efficacy and potentially reduce required doses, enhancing safety and minimizing antibiotic resistance concerns. 4. Long-Term Outcomes and Resistance: The long-term effects of inhaled antibiotics on lung function, antibiotic resistance development, and other relevant outcomes require further investigation. Additionally, exploring the potential for emergence of resistant strains specific to inhaled antibiotics is crucial for informing sustainable resistance management strategies.

## Conclusions

Tobramycin or colistin may have a positive impact on the clinical and microbiological cure rates of ventilator-associated pneumonia. The quality of evidence is low indicating a high degree of uncertainty. This highlights the importance of conducting more rigorous and well-designed studies to improve the quality of evidence and inform clinical decision-making.

### Supplementary Information

**Supplementary Material 1.**

## Data Availability

The data that support the findings of this study are available from the corresponding author upon reasonable request.
