# Supplementary material for: Efficacy of adjunctive inhaled colistin and tobramycin for ventilator-associated pneumonia: systematic review and meta-analysis
Source: BMC Pulm Med. 2024 May 2;24:213. doi: 10.1186/s12890-024-03032-7 (PMC11064396; doi:10.1186/s12890-024-03032-7)
Supplement: Supplementary file 1 — Supplementary Material 1. [file 12890_2024_3032_MOESM1_ESM.docx]

**Table S1Characteristic of included study**

| **Study** | **Sample size**  **(Treatment /Control)** | **Mean of Age in years (Treatment group /Control group)** | **APACHE II(Treatment group /Control group)** | **Diagnostic criteria of Ventilator-associated pneumonia;** | **Isolated bacteria (%), distribution of various bacterial species.** | **Therapy(T/C) AND**  **Percentage of colistin/tobramycin used, duration of interventions** |
| --- | --- | --- | --- | --- | --- | --- |
| Brown,1990(1) | 25/16 | 57.3 years /58.4 years  No other detail of distribution of age provided | / | (i) roentenogram (ii) Gram stain of endotracheal secretions (iii)grossly purulent tracheobronchial secretions, and (iv)note **a** | P.Aeruginosa (18,44%),  Klebsiella-Enterobacter (13,32%)  Serratia-Citrobacter species (6,15%),  other nonfermenters accounted (4,10%) | Intervention group: Tobramycin 40mg q8h endotracheal (100% received this treatment ) + Intravenous tobramycin + cefazolin or piperacillin when organism resistant to cefazolin was  either clinically suspected or proven by cultures. (28% and 72% respectively received this treatments)  Control group: Placebo + Intravenous tobramycin + cefazolin or piperacillin when organism resistant to cefazolin was  either clinically suspected or proven by cultures. (35% and 65% respectively received this treatments) |
| Le Conte P,2000(2) | 21/17 | No other detail of distribution of age provided | No provided. | No provided. | Pseudomonas (53%),  Haemophilus (20%),  Enterobacter (13%),  E. coli (10%),  Klebsiella (3%) | Tobramycin 6mg/kg/d  (+systematic β-lactam/tobramycin) x 5 days. **Percentage of colistin/tobramycin used; duration of interventions no provided** |
| Rattanaumpawan,2010(3) | 51/49 | 70.2 years /66.2 years  No other detail of distribution of age provided | 19/18 | (i) mechanical ventilation≥48h (ii)fever (iii)leucocytosis (iv)purulent tracheal secretions (v)new or progressive pulmonary infiltrates (vi)Gram negative bacilli were isolated from an endotracheal tube aspirate specimen. | Acinetobacter (50%),  Pseudomonas (26%),  Klebsiella (15%),  E. coli (5%),  Enterobacter (2%),  Stenotrophomonas (2%) | Intervention group:  Colistin nebulized 75mg q12h x 9 days (duration of nebulized therapy 12 days) +  systematic antibiotic (imipenem or meropenem 31%, colistin 35%, piperacillin/tazobactam 9.8%, cefoperazone/sulbactam 9.8%, ceftazidime or cefepime or ceftriaxone 5.9%, others 22%)  Control group  ±4.6/ Sterile normal saline nebulized (duration of nebulized therapy 13 days) + ( imipenem or meropenem 40%, colistin 20%, piperacillin/tazobactam 20%, cefoperazone/sulbactam 10%, ceftazidime or cefepime or ceftriaxone 6%, others 10%) |
| Nassar,2018(4) | 52/50 | 55.5±17 years (this value corresponds to all patients, details by group no was provided) | 15.42±7.91 | (i)CPIS＞6 (ii)Microbiological culture | Klebsiella spp. (31%),  Pseudomonas spp. (38%),  Acinetobacter spp. (31%) | Intervention group: Colistin 1 million IU(≈33.3mg CBA) q8h/Blank x 5 days +  +β-lactam-based antibiotic with antipseudomonal  activity (piperacillin-tazobactam, cefepime, or  meropenem) plus a non-β-lactam-based antibiotic  with antipseudomonal activity (levofloxacin) plus an  antibiotic with MRSA activity (vancomicin or  linezolide). **Percentage of colistin/tobramycin used; duration of interventions no provided**  Control group: β-lactam-based antibiotic with antipseudomonal  activity (piperacillin-tazobactam, cefepime, or  meropenem) plus a non-β-lactam-based antibiotic with antipseudomonal activity (levofloxacin) plus an antibiotic with MRSA activity (vancomycin or linezolid). **Percentage of colistin/tobramycin used; duration of interventions no provided** |
| Stokker 2020(5) | 13/13 | 58 years with IQR between 42 to 49 years / 59 years with a IQR between 43 to 66 years. No other detail of distribution of age provided. | 21/14 | (i)Mechanical ventilation≥48 h  (ii)New or progressive radiologic pulmonary infiltrate  (iii)at least two of the following three criteria (< 24 h): **e** | Enterobacter (11%),  Staphylococcus aureus (44%)  Escherichia coli (33%)  Stenotrophomonas maltophilia  & Enterococcus faecalis (11%) | Intervention group:  Tobramycin inhalation 300mg x 8 days +systematic antibiotics (no detail of systemic antibiotics used were provided)  Control group: Placebo inhalation bid + systematic antibiotics (no detail of systemic antibiotics used were provided) |
| Hallal,2007(6) | 5/5 | 52.6 years (range 23 to 72 years) / 53.6 (range 24 to 28 years). No other detail of distribution of age provided | 17/ 15 | New or progressive/persistent infiltrate,  purulent tracheal secretion, and a positive  bronchoalveolar lavage (BAL) showing 104 bacterial  colony-forming units (CFU)/mL in the  recovered fluid | Pseudomonas (60%),  Acinetobacter (20%),  Staphylococcus (20%) | Intervention group:  1–IV placebo q24 h + tobramycin inhalation  300 mg , 5 mL q12 h + β-lactam antibiotic (piperacillin/tazobactam or imipenem/cilastatin). **Percentage of colistin/tobramycin used; duration of interventions no provided** Vancomycin was allowed if necessary for gram-positive coverage.  Control group: IV tobramycin q24 h +placebo nebulization, 5 mL q12 h a -lactam antibiotic (piperacillin/tazobactam or imipenem/cilastatin). **Percentage of colistin/tobramycin used; duration of interventions no provided** Vancomycin was allowed if necessary for gram-pos-  itive coverage. |
| Angermair 2023(7) | 14/14 | 64 years with IQR between 56 to 72 years  / 65 with IQR between 57 to 70 years. No other detail of distribution of age provided | 24/ 21 | VAP was defined as the presence  of a new or progressive pulmonary infiltrate in chest radiography  or CT scan and two of the following:  1) Temperature >38.3 8C or <36.0 8C,  2) Leukocyte count >12.000/mL or <4.000/mL,  3) Purulent tracheal secretions | Serratia marcescens (23%)  Pseudomonas aeruginosa (27%)  Enterobacter cloacae (15%)  Klebsiella pneumoniae (15%)  Escherichia coli (19%) | Intervention group:  300 mg of aerosolized Tobramycin administered twice daily for five days +systematic antibiotics (Carbapenem: 8.82%  Penicillin/beta-lactamase inhibitor: 26.47%  Fluoroquinolone: 17.65%  Cephalosporin: 5.88%  Combination therapy: 17.65%  Monotherapy: 23.53%)  Control group: 5 mL of aerosolized 0.9% sodium chloride solution + systematic antibiotics (Carbapenem: 17.24%  Penicillin/beta-lactamase inhibitor: 20.69%  Fluoroquinolone: 17.24%  Cephalosporin: 3.45%  Combination therapy: 17.24%  Monotherapy: 24.14%) |

**Table S2. Clinical and microbiological cure criteria in included studies.**

| **Study** | **Inhalation device** | **Prior antibiotics use** | **Clinical cure criteria** | **Microbiological cure criteria** |
| --- | --- | --- | --- | --- |
| Brown,1990(1) | small-bore plastic cannula threaded down the endotracheal or tracheostomy tube | The length of prior use is unknown | Patients were deemed clinically improved if disappearance of fever and improvement.  of pulmonary infiltrates and physical findings of pneumonia were noted. "Relapse" required a satisfactory initial clinical response followed by clinical and radiographic deterioration.  "Failure" was defined as lack of clinical and  radiographic improvement over a reasonable period, typically 5 to 7 days. | Bacteriologic outcome was assessed by disappearance of the offending pathogen(s), altered susceptibilities of the organisms recovered, and the emergence of new potential pathogens during therapy. "Pathogen eliminated" implied that follow-up cultures failed to demonstrate the initial causative bacterium. "Recurrence" was defined as initial disappearance and then reappearance of the causative pathogen(s). Bacteriologic failure required ongoing.  demonstration of the initial pathogen |
| Le Conte P,2000(2) | pneumatic nebulizer | The length of prior use is unknown | Extubation within 10 days | NA |
| Rattanaumpawan,2010(3) | jet/ultrasonic nebulizer | 72.5%/79.6% prior use of antibiotic(s) within 72 h | Complete resolution of all signs and symptoms  of pneumonia, and improvement or lack of  progression of all abnormalities on the chest  radiograph | Eradication or presumed eradication.  after antimicrobial treatment |
| Nassar,2018(4) | jet nebulizer | The length of prior use is unknown | the CPIS was calculated on day 6 to  determine the clinical outcome. A CPIS less  than 6 was interpreted as clinical improvement | Clearance: no growth; Resistance: persistent growth; Superinfection: eradication of the original pathogen, but growth of a new pathogen; and Resistance and superinfection: persistent growth of original plus new pathogen |
| Stokker 2020(5) | commercial device | The length of prior use is unknown | Nonresponse is considered when at least one of the following criteria is present:  (1) No improvement of the arterial O2 tension to inspired O2 fraction ratio  (2) Persistence of fever (≥38°C) or hypothermia (<35.5°C) together with purulent  respiratory secretions  (3) increase in the pulmonary infiltrates on chest radiograph of greater than or equal to 50%  (4) occurrence of septic shock or multiple organ dysfunction syndrome, defined as three or more organ system failures do not present on Day 1 | Eradication or presumed eradication.  after antimicrobial treatment |
| Hallal,2007(6) | Jet nebulizer | Duration of empiric treatment before randomization in T :2.2 ± 1.3 and C: 1.9 ± 2.10 | Extubation within the study period, improving of MODS, resolution of fever, pulmonary infiltrates, and physical signs of pneumonia. | NA |
| Angermair 2023(7) | commercial device | The length of prior use is unknown | Clinical cure of pneumonia assessed each day until two days after the termination of systemic antibiotic treatment, | eradication of the endobronchial  Gram-negative bacteria in the pulmonary system at visit 6 of the  treatment. |

T：experimental group, C：placebo or control group, APACHE II：Acute Physiology and Chronic Health Evaluation (APACHE) II score, CPIS：clinical pulmonary infection score, MDR：multiple drugs resistant, XDR：extensively drug resistant, PDR：pan-drug resistant, IH：inhaled, HAP：hospital acquired pneumonia，VAP：ventilator-associated pneumonia，, MDRO：multi-drug resistant organism

**References**:

1. Brown RB, Kruse JA, Counts GW, Russell JA, Christou NV, Sands ML. Double-blind study of endotracheal tobramycin in the treatment of gram-negative bacterial pneumonia. The Endotracheal Tobramycin Study Group. Antimicrob Agents Chemother. 1990;34(2):269-72.

2. Le Conte P, Potel G, Clementi E, Legras A, Villers D, Bironneau E, et al. [Administration of tobramycin aerosols in patients with nosocomial pneumonia: a preliminary study]. Presse Med. 2000;29(2):76-8.

3. Rattanaumpawan P, Lorsutthitham J, Ungprasert P, Angkasekwinai N, Thamlikitkul V. Randomized controlled trial of nebulized colistimethate sodium as adjunctive therapy of ventilator-associated pneumonia caused by Gram-negative bacteria. J Antimicrob Chemother. 2010;65(12):2645-9.

4. Nassar YS, Saber-Ayad M, Shash RY. Combined microbiological and clinical outcomes of short-term inhaled colistin adjunctive therapy in ventilator-associated pneumonia. The Egyptian Journal of Chest Diseases and Tuberculosis. 2018;67(4).

5. Stokker J, Karami M, Hoek R, Gommers D, van der Eerden M. Effect of adjunctive tobramycin inhalation versus placebo on early clinical response in the treatment of ventilator-associated pneumonia: the VAPORISE randomized-controlled trial. Intensive Care Med. 2020;46(3):546-8.

6. Hallal A, Cohn SM, Namias N, Habib F, Baracco G, Manning RJ, et al. Aerosolized tobramycin in the treatment of ventilator-associated pneumonia: a pilot study. Surg Infect (Larchmt). 2007;8(1):73-82.

7. Angermair S, Deja M, Thronicke A, Grehn C, Akbari N, Uhrig A, et al. A prospective phase IIA multicenter double-blinded randomized placebo-controlled clinical trial evaluating the efficacy and safety of inhaled Tobramycin in patients with ventilator-associated pneumonia (iToVAP). Anaesth Crit Care Pain Med. 2023;42(5):101249.
